# Gravity Compensation Using EGM2008 for High-Precision Long-Term Inertial Navigation Systems

**DOI:** 10.3390/s16122177

**Published:** 2016-12-18

**Authors:** Ruonan Wu, Qiuping Wu, Fengtian Han, Tianyi Liu, Peida Hu, Haixia Li

**Affiliations:** Department of Precision Instrument, Tsinghua University, Beijing 100084, China; wrn13@mails.tsinghua.edu.cn (R.W.); liuty14@mails.tsinghua.edu.cn (T.L.); hpd211@mail.tsinghua.edu.cn (P.H.); li-hx03@mails.tsinghua.edu.cn (H.L.)

**Keywords:** gravity disturbance vector compensation, high-precision INS, EGM2008, deflections of the vertical (DOV), update interval

## Abstract

The gravity disturbance vector is one of the major error sources in high-precision and long-term inertial navigation applications. Specific to the inertial navigation systems (INSs) with high-order horizontal damping networks, analyses of the error propagation show that the gravity-induced errors exist almost exclusively in the horizontal channels and are mostly caused by deflections of the vertical (DOV). Low-frequency components of the DOV propagate into the latitude and longitude errors at a ratio of 1:1 and time-varying fluctuations in the DOV excite Schuler oscillation. This paper presents two gravity compensation methods using the Earth Gravitational Model 2008 (EGM2008), namely, interpolation from the off-line database and computing gravity vectors directly using the spherical harmonic model. Particular attention is given to the error contribution of the gravity update interval and computing time delay. It is recommended for the marine navigation that a gravity vector should be calculated within 1 s and updated every 100 s at most. To meet this demand, the time duration of calculating the current gravity vector using EGM2008 has been reduced to less than 1 s by optimizing the calculation procedure. A few off-line experiments were conducted using the data of a shipborne INS collected during an actual sea test. With the aid of EGM2008, most of the low-frequency components of the position errors caused by the gravity disturbance vector have been removed and the Schuler oscillation has been attenuated effectively. In the rugged terrain, the horizontal position error could be reduced at best 48.85% of its regional maximum. The experimental results match with the theoretical analysis and indicate that EGM2008 is suitable for gravity compensation of the high-precision and long-term INSs.

## 1. Introduction

In inertial navigation systems (INSs), the accelerometer-sensed specific force consists of the kinematic acceleration and the gravitational acceleration. Thus gravitational information along the route plays an important role to extract the kinematic acceleration of the vehicle first. Generally, the normal gravity is employed in order to achieve a balance between the accuracy and computation efficiency. The difference between the actual and the normal gravity vector, namely the so-called gravity disturbance vector, is one of the error sources in INSs. The influence of the gravity disturbance vector is mostly negligible compared to inertial measurement unit (IMU) errors and some other factors. However, for the long-term INSs with precise inertial sensors and efficient algorithms, the gravitational errors should be taken into account to achieve high navigation precision. A number of researchers have used the covariance propagation analysis to theoretically investigate the gravity induced navigation errors as early as the second half of the 20th century. Levine and Gelb evaluated the gravity-induced velocity, position, azimuth and platform tilt errors for a wide range of vehicle speeds, and concluded that they were significantly affected by mission speed, operating latitude and system damping ratio [1]. Schwarz’s results showed that the gravity-induced position errors mainly resulted from the poorly modeled deflections of the vertical (DOV), while the effect of the gravity anomaly and geoidal undulations could be in general neglected [2]. Harriman and Harrison reported that errors in a damped INS were predominately cross-track at all times, and that the position and velocity errors resulting from gravity disturbance errors would decrease if the flight speed or altitude was increased [3]. Soon it was observed that on each of several westbound transpacific flights the Schuler oscillation within the velocity errors would grow rather significantly along the Kuril trench [4], which provided some evidences for the theoretical works. Besides the pure-inertia systems, even though most errors can be corrected by the Global Positioning System (GPS) updates in a GPS/INS integrated system, it should be noted that the gravity-induced attitude errors still exist [5,6] and that position and velocity errors would arise during the time of GPS signal loss [7]. Therefore, the compensation of the gravity disturbance vector is imperative for those applications demanding high-accuracy inertial navigation solutions, such as submarines.

Statistic models can be used not only to analyze the error propagation of unknown gravity, but also for optimal prediction and filtering of the gravity disturbance in INSs [5], airborne gravimetry [8] and precise orbit determination for Earth-orbiting satellites [9]. The third-order Markov undulation model is reported to be both convenient and appropriate for the analysis of the gravity uncertainties induced errors in INSs, and suitable for the Kalman filter technique [10]. However, the statistic models of the gravity disturbance vector have two inherent disadvantages. First, a single low-order model is not sufficient to describe a large-scale gravity field due to the diversity of topography; and secondly, a large amount of a priori information must be collected to precisely estimate the parameters of the statistic models. Nowadays, benefiting from advanced gravimeters and gradiometers and easier access to accurate high-resolution gravity data archives, it has become a better option using the attainable gravitational information directly. Several theoretical works and simulations indicated that the gradiometer would help improve the navigation performance with in situ measurements of the gravity field [11,12], yet the high cost still limits its application in comparison to accurate high-resolution data archives. According to Kwon and Jekeli’s research, with ground data gridded with 2 arc-minutes resolution and accurate to better than 3 mGal, the error in gravity compensation contributes less than 5 m to the position error after one hour of free-inertial navigation for a typical flight trajectory at 5 km altitude and 300 km/h speed [13]. A few global or near-global maps of DOV and gravity disturbances have already been released, which are adequate to meet such requirement. For instance, the DOV data set released under the model GGMplus has a much higher resolution of 7.2 arc-seconds, covering 80% of Earth’s land masses [14]. To make the best of such ground data, the methods of interpolation and upward continuation must be carefully chosen [13,15].

Another alternative to obtain the gravitational information, which is chosen in this work, is to make use of spherical harmonic models. The Earth Gravitational Model 2008 (EGM2008) is such a model developed by a least squares combination of the satellite-only ITG-GTACE03S gravitational model with a global set of area-mean free-air gravity anomalies [16]. Assessments in various regions around the world indicate that it performs comparably with contemporary detailed regional geoid models [17,18,19,20,21]. For example, the EGM2008 DOVs over USA, Europe and Australia are within 1.1 to 1.3 arc-seconds of independent astrogeodetic values [16,17]. This model represents significant improvements by a factor of six in resolution, and by factors of three to six in accuracy over its predecessor EGM96 that is inadequate for very precise navigation [16,22]. Thus it is reasonable to take advantage of the EGM2008 for gravity compensation in INSs. A few studies based on the theoretical analysis and simulation on such methods indicate that it is effective to compensate gravity induced INS errors with the aid of EGM2008 [5,23,24,25].

As the errors of free-inertial navigation diverge over time, most INSs utilize a kind of feedback loop with external altitude and velocity references to restrain the divergence in the vertical channel and the Schuler oscillation in the horizontal channels, namely the damping network. Existing studies basically aim at short-term free-inertial navigation or INSs with simple damping networks, while this paper focuses on the high-precision and long-term INSs with high-order damping networks. First, the error propagation of the gravity disturbance vector in such systems is analyzed and two methods for gravity compensation using the EGM2008 are provided. Then a formula is developed to characterize the compensation error resulting from the gravity update interval and the computing time delay, which can provide some references for the implementation of real-time gravity compensation. Correspondingly, the computation burden of gravity vectors using the high degree and order spherical harmonic model is reduced by investigating and optimizing the calculation procedure. Finally, a few off-line experiments using the data from an actual sea test are presented to validate the theoretical analysis and simulation results.

## 2. Gravity Disturbance Vector Induced Position Errors

### 2.1. Error Propagation

**Figure 1:**
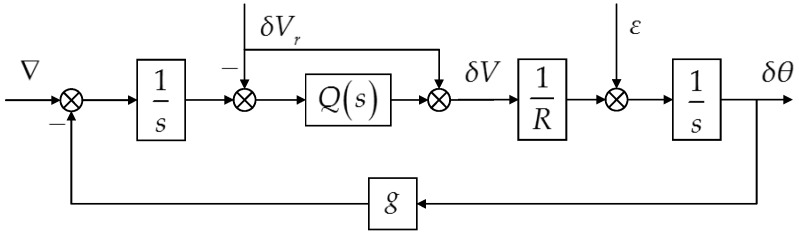
Error propagation of the generalized damped Schuler loop.

To maintain long-term precise navigation, INSs must introduce external altitude and velocity references to provide suitable damping for the vertical channel and the Schuler loops. Under this circumstance, errors in the horizontal channels are more concerned than the vertical one. Ignoring the cross-coupling with the vertical channel and the cross-coupling with the Earth’s rotation rate, Figure 1 illustrates the propagation of typical error sources, including the gyro bias ε, the accelerometer bias ∇ and the reference velocity error ${\delta V}_{r}$, in the generalized damped Schuler loop [25].

Here, g and R are the local gravity and the average radius of the Earth, respectively. The velocity error and platform tilt are represented by δV and δθ. The INSs discussed in this paper adopt a kind of high-order damping network, which is designed based on the complementary filtering to obtain 40 dB/10 dec or higher attenuation rate to both low-frequency and high-frequency reference velocity errors [26]. The transfer function of such a damping network, Q(s), is given by:

(1) $$Q\left(s\right)=\frac{\left\{\begin{array}{l} 2\mu s^{2}\left(s^{2}+2\mu \omega_{s}s+\omega_{s}^{2}\right)+\\ \left(\omega_{s}s+2\mu \omega_{s}^{2}\right)\left[\left(1+4\zeta \mu \right)s^{2}+2\left(\zeta +\mu \right)\omega_{s}s+\omega_{s}^{2}\right] \end{array}\right\}}{\left\{\begin{array}{l} 2\mu s^{2}\left[s^{2}+2\left(\zeta +\mu \right)\omega_{s}s+\left(1+4\zeta \mu \right)\omega_{s}^{2}\right]+\\ 4\zeta \mu \omega_{s}^{3}s+\left(\omega_{s}s+2\mu \omega_{s}^{2}\right)\left[s^{2}+2\left(\zeta +\mu \right)\omega_{s}s+\omega_{s}^{2}\right] \end{array}\right\}}$$

where $\omega_{s}=\sqrt{g/R}$ is the Schuler angular frequency, and ζ and μ are two coefficients determining the attenuation response. The values for μ and ζ have been optimized and are assigned μ = 0.5 and ζ = 1.296 in our shipborne INS to obtain the required attenuation rate mentioned above. The analysis of the gravity disturbance vector induced position errors will be based on these values.

### 2.2. Gravity Disturbance Vector and Its Induced Position Errors

The accelerometer sensed specific force vector ***f*** is the combination of the kinematic acceleration vector ***a*** and the gravitational acceleration vector ***G***, as:

(2) f=a−G

Considering the centrifugal effect of the Earth rotation, Equation (2) can be rewritten in the following form:

(3) $$a=f+g+\omega_{ie}\times \left(\omega_{ie}\times r\right)$$

where ***g*** is the gravity vector and $\omega_{ie}$ is the Earth’s rotation vector. The radius vector ***r*** defines the position to the Earth’s center of mass. Obviously it can be seen from Equations (2) and (3) that INSs need gravitational information to extract the kinematic acceleration of the vehicle. The normal gravity model is frequently employed because it can meet the accuracy requirement in most cases and is both simple and convenient to be calculated. This model is based on an ellipsoid of revolution having the same mass and rotation rate with the Earth, namely the so-called reference ellipsoid. As the normal gravity vector γ is perpendicular to the surface of the reference ellipsoid, its vertical component equals its magnitude γ, as:

(4) $$\gamma^{n}={\left(\begin{array}{ccc} 0 & 0 & \gamma \end{array}\right)}^{T}$$

where the superscript *n* indicates the vector in the navigation coordinate system (n-frame).

Since both the shape and mass distribution of the Earth are not ideal, there exists difference between the actual and the normal gravity vector at the same position. This difference is called the gravity disturbance vector, expressed in n-frame as:

(5) $$\delta g^{n}={\left(\begin{array}{ccc} -\xi g & -\eta g & \delta g \end{array}\right)}^{T}$$

where ξ and η are the north and the west component of DOV respectively, which represent the difference between the orientations of the actual and the normal gravity vector. δg is the magnitude of the gravity disturbance vector, called the gravity disturbance.

The gravity disturbance vector barely affects the vertical channel damped by the external altitude reference input, thus we can focus on the latitude and longitude errors only. According to Figure 1, the accelerometer error induced horizontal position error δr is given in:

(6) $$\delta r\left(s\right)=R\delta \theta \left(s\right)=\frac{1}{\omega_{s}^{2}}I\left(s\right)\nabla \left(s\right)$$

where:

(7) $$I\left(s\right)=\frac{\omega_{s}^{2}Q\left(s\right)}{s^{2}+\omega_{s}^{2}Q\left(s\right)}$$

It can be concluded from Equation (3) that the gravity disturbance vector has the same propagation with the accelerometer error. Replacing ∇ in Equation (6) with the horizontal components of Equation (5), namely ξg and ηg, yields their induced latitude and longitude errors:

(8) $$\begin{array}{l} \delta L\left(s\right)=\frac{\omega_{s}^{2}Q\left(s\right)}{s^{2}+\omega_{s}^{2}Q\left(s\right)}\xi \left(s\right)\\ \delta l\left(s\right)\cos\left(L\right)=\frac{\omega_{s}^{2}Q\left(s\right)}{s^{2}+\omega_{s}^{2}Q\left(s\right)}\eta \left(s\right) \end{array}\}$$

where *L* and *l* represent the latitude and the longitude, respectively.

**Figure 2:**
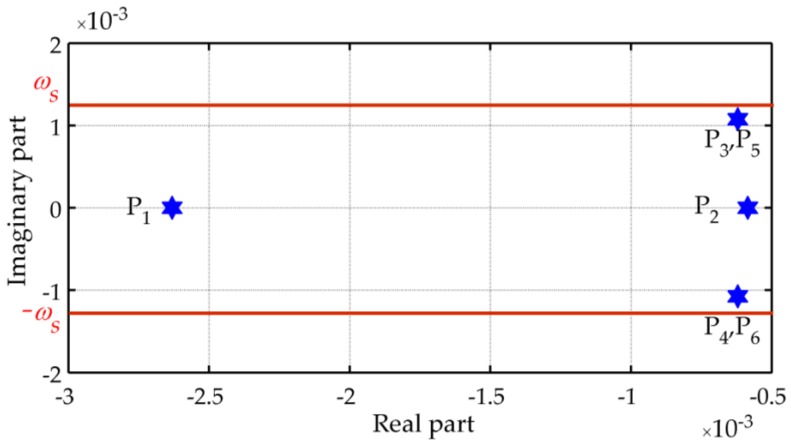
Pole plot of I(s) (μ = 0.5, ζ = 1.296).

**Figure 3:**
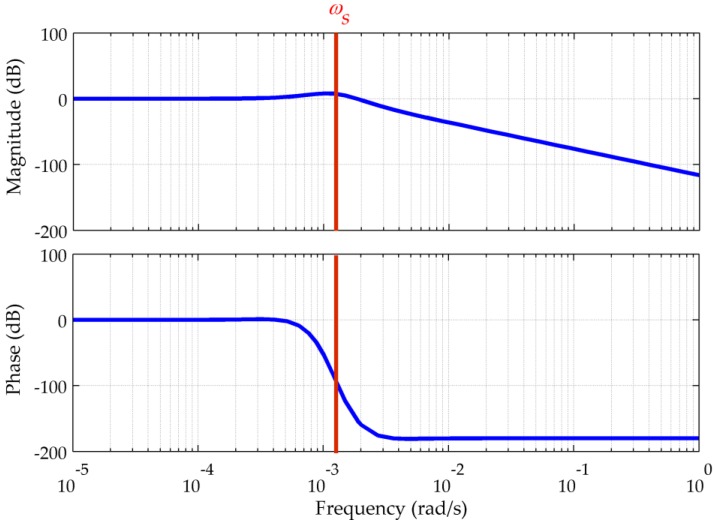
Bode plot of I(s) (μ = 0.5, ζ = 1.296).

In other words, δL(s) and δl(s)cos(L) are the responses of a linear system to the corresponding components of the DOV, whose transfer function is I(s). Using Equations (1) and (7), we can draw the pole plot and the Bode plot of I(s), as shown in Figure 2 and Figure 3, respectively.

Figure 2 shows that all of the poles have negative real parts and that four of them are complex with imaginary components near the Schuler angular frequency. This means that the system is stable but has underdamped transient responses similar to the Schuler oscillation. The stable Schuler loop acts as a low-pass filter, whose detailed frequency response has been illustrated in Figure 3. From the Bode plot it can be concluded that the gravity disturbance vector induced latitude and longitude errors consist of two parts.

First, at low frequencies there are no amplitude or phase distortions, hence ξ and η, and their induced δL(s) and δl(s)cos(L) share the same low-frequency components, respectively. Secondly, the peak around the Schuler angular frequency indicates that δL(s) and δl(s)cos(L) also include underdamped Schuler oscillations with amplitude related to the fluctuations of ξ and η. Since the global maximum of the DOV is more than 100 arc-seconds, for high-precision and long-term INSs the resulting errors cannot be neglected, and must be carefully compensated. Besides, Figure 3 shows that there is a significant attenuation, e.g., higher than 30 dB at angular frequencies above 0.01 rad/s, which corresponds a spatial wavelength of 6.28 km for a speed of 10 m/s. As the spatial frequency of the DOV is fixed, higher speed means faster change with the time. Thus, the gravitational data used in such a system do not require extremely high spatial resolution because of the low-pass characteristic.

## 3. Gravity Compensation Using a Spherical Harmonic Model

Assuming that the density outside the Earth is zero, the gravitational potential *V* satisfies the Laplace equation and can be expressed by a harmonic function. At a position defined by its geocentric distance *r*, geocentric co-latitude φ (defined as 90°-latitude) and longitude *l*, *V* is given by [27]:

(9) $$V(r,\varphi ,l)=\frac{KM}{r}\sum_{n=0}^{\infty }\sum_{m=0}^{n}{\left(\frac{a}{r}\right)}^{n}({\bar{C}}_{nm}\cos ml+{\bar{S}}_{nm}\sin ml)\cdot {\bar{P}}_{nm}(\cos\varphi )]$$

where *KM* is the geocentric gravitational constant, *a* is the semi-major axis of the reference ellipsoid, ${\bar{C}}_{nm}$ and ${\bar{S}}_{nm}$ are fully-normalized, unit-less, spherical harmonic coefficients, and ${\bar{P}}_{nm}(\cos\varphi )$ is the fully normalized associated Legendre function (ALF) of the first kind, of degree *n* and order *m*. Gravitational acceleration is the gradient vector of the gravitational potential, of which each component deriving from Equation (9) is given by [27]:

(10) $$\begin{array}{ll} G_{r} & =\frac{\partial V(r,\varphi ,l)}{\partial r}\\ & =-\frac{KM}{r^{2}}[1+\sum_{n=2}^{N\max}(n+1){\left(\frac{a}{r}\right)}^{n}\sum_{m=0}^{n}\left({\bar{C}}_{nm}\cos ml+{\bar{S}}_{nm}\sin ml)\cdot {\bar{P}}_{nm}(\cos\varphi \right)]\\ G_{\varphi } & =\frac{\partial V(r,\varphi ,l)}{r\partial \varphi }\\ & =\frac{KM}{r^{2}}\sum_{n=2}^{N\max}{\left(\frac{a}{r}\right)}^{n}\sum_{m=0}^{n}({\bar{C}}_{nm}\cos ml+{\bar{S}}_{nm}\sin ml)\cdot \frac{\partial {\bar{P}}_{nm}(\cos\varphi )}{\partial \varphi }\\ G_{l} & =\frac{\partial V(r,\varphi ,l)}{r\sin\varphi \partial l}\\ & =\frac{KM}{r^{2}\sin\varphi }\sum_{n=2}^{N\max}{\left(\frac{a}{r}\right)}^{n}\sum_{m=0}^{n}m(-{\bar{C}}_{nm}\sin ml+{\bar{S}}_{nm}\cos ml)\cdot {\bar{P}}_{nm}(\cos\varphi ) \end{array}\}$$

Then the transformation to the n-frame can be written in the form:

(11) $$G^{n}=\left(\begin{array}{ccc} -\sin L & 0 & \cos L\\ 0 & 1 & 0\\ -\cos L & 0 & -\sin L \end{array}\right)\left(\begin{array}{ccc} \cos\varphi & 0 & \sin\varphi \\ 0 & 1 & 0\\ -\sin\varphi & 0 & \cos\varphi \end{array}\right)\left(\begin{array}{c} G_{\varphi }\\ G_{l}\\ G_{r} \end{array}\right)$$

Finally, the Earth’s gravity consisting of the gravitational acceleration and the centrifugal acceleration of the Earth’s rotation can be expressed as:

(12) $$g^{n}=G^{n}+\left(\begin{array}{c} -\omega_{ie}^{2}(R_{N}+h)\cos L\sin L\\ 0\\ -\omega_{ie}^{2}(R_{N}+h)\cos^{2}L \end{array}\right)$$

where $\omega_{ie}$ is the Earth’s rotation rate and *h* is the altitude. $R_{N}$ is the normal radius of curvature taken in the direction of the prime vertical, given in:

(13) $$R_{N}=\frac{a}{{\left(1-e^{2}\sin^{2}L\right)}^{1/2}}$$

where *e* is the first eccentricity of the reference ellipsoid.

EGM2008 provides a set of estimated spherical harmonic coefficients, up to degree 2190 and order 2159 [28]. Then we can use Equations (10)–(13) to calculate the gravity vector at any given position on or outside the Earth. Since the ultra-high degree and order ALFs in Equation (10) could range over thousands of orders of magnitude, we have to use some special techniques to avoid underflow and overflow problems when computing them. Existing algorithms shows different performance in numerical stability and accuracy, and it is common in geodesy to use Clenshaw’s method. Here we will utilize the modified forward column method because it is equivalent to Clenshaw’s method in both efficiency and precision, while the mechanisms within the computation process are highly intuitive and transparent, and also because it can output individual values of ALFs and their first derivatives [29].

The only problem left here is how to implement real-time gravity compensation. There are two options: (1) compute the EGM2008 and record an offline database for real-time interpolation; and (2) compute the EGM2008 directly in situ. Generally, the first one is preferred because the calculation of the spherical harmonic model, to ultra-high degree and order, is thought to be complicated and time-consuming and thus a huge burden for INSs. However, with analysis and optimization, we have found that the second choice can also satisfy the requirement of real-time compensation. This will be discussed in detail in the next section.

## 4. Real-Time Gravity Compensation

### 4.1. Time Requirements for Real-Time Compensation

A test result on a digital signal processor (DSP) showed that spherical harmonic models of degree 12 are applicable to low- and middle-precision INSs with update frequencies less than 400 Hz [25,30]. To further improve the spatial resolution and reduce the computational complexity, a low-order polynomials was used to approximate the spherical harmonic model in a small area and showed good performance for real-time free-inertial solutions [24]. However, it is actually not necessary to update the gravity data that frequently because they change much slower than the typical IMU outputs. And a spherical harmonic model of degree 12 is obviously not suitable for the high-precision and long-term inertial navigation. Therefore, a new time requirement is developed in this section.

**Figure 4:**
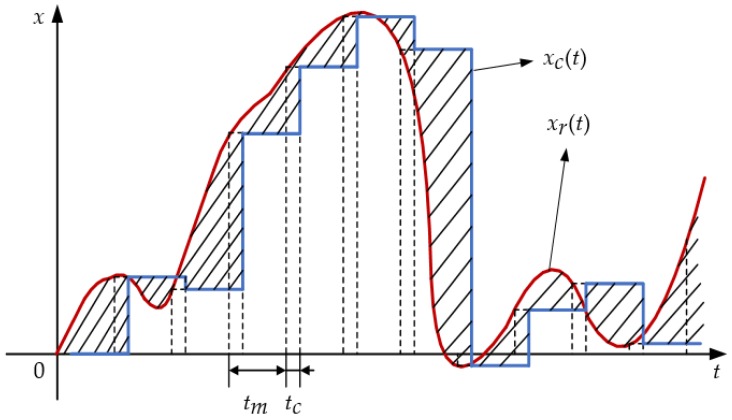
Effect of gravity update interval and computing time delay on compensation accuracy.

It has been concluded above that it is mainly the low and medium frequency components of the DOV that propagate into the position errors, which suggest that we can safely lengthen the time interval of gravity updating. In addition, the time spent on interpolation from database and calculation using the EGM2008 delays the values’ update, although the first one is too fast to be observed. The compensation error resulting from the gravity update interval $t_{m}$ and computing time delay $t_{c}$ is illustrated in Figure 4.

Although in practice $t_{c}$ might change within a small range, it can be assumed constant for simplicity. During the navigation process, the computation processes of navigation solutions and gravity data are concurrent. Gravity calculation is triggered at a constant interval $t_{m}$, and the gravity vector will be maintained at its current value until the gravity calculation process outputs a new one after a time delay $t_{c}$. Such arrangement guarantees that the gravity calculation does not interrupt the navigation process.

In Figure 4, $x_{r}(t)$ and $x_{c}(t)$ denote the truth and actually used values of the gravitational information. The root mean square (RMS) of their discrepancy (denoted by the hatched areas in Figure 4) is given by:

(14) $$W^{2}=\underset{T\to \infty }{\lim}\frac{1}{T}\int_{-\frac{T}{2}}^{\frac{T}{2}}{\left[x_{c}\left(t\right)-x_{r}\left(t\right)\right]}^{2}dt$$

As $x_{c}(t)$ has a staircase shape, Equation (14) can be written in the form of piecewise integrations:

(15) $$W^{2}=\underset{N\to \infty }{\lim}\frac{1}{2N+1}\sum_{k=-N}^{N}\frac{1}{t_{m}}\int_{0}^{t_{m}}{\left[x_{r}\left(kt_{m}-t_{c}\right)-x_{r}\left(kt_{m}+t\right)\right]}^{2}dt$$

Expanding Equation (15) and interchanging the order of summation and integration, we have:

(16) $$\begin{array}{ll} W^{2} & =\underset{N\to \infty }{\lim}\frac{1}{2N+1}\sum_{k=-N}^{N}x_{r}^{2}\left(kt_{m}-t_{c}\right)+\underset{T\to \infty }{\lim}\frac{1}{T}\int_{-\frac{T}{2}}^{\frac{T}{2}}x_{r}^{2}\left(t\right)dt\\ & -2\underset{N\to \infty }{\lim}\frac{1}{t_{m}}\int_{0}^{t_{m}}\frac{1}{2N+1}\sum_{k=-N}^{N}x_{r}\left(kt_{m}+t\right)x_{r}\left(kt_{m}-t_{c}\right)dt \end{array}$$

If the sample frequency criterion is satisfied, that is, $t_{m}<1/\left(2f_{max}\right)$, the discrete sequence of samples $x_{r}\left(kt_{m}-t_{c}\right)$ are able to capture all the information from the continuous-time signal $x_{r}(t)$. Then the first term in the right-hand side of Equation (16) equals the second one. Moreover, as the element to be integrated in the last term is bounded for every *N*, according to the dominated convergence theorem we can also interchange the order of limit and integration and get the integral of the autocorrelation function. Thus Equation (16) can be finally written in the following form:

(17) $$W^{2}=2\Phi_{r}\left(0\right)-2\frac{1}{t_{m}}\int_{0}^{t_{m}}\Phi_{r}\left(\tau +t_{c}\right)d\tau$$

where $\Phi_{r}(\tau )$ is the autocorrelation of $x_{r}(t)$.

Local gravity field can be characterized by exponential correlation function [1], such as:

(18) $$\Phi_{r}\left(\tau \right)=\sigma_{r}^{2}e^{-d\left|\tau \right|}$$

where $\sigma_{r}^{2}$ is the variance of $x_{r}(t)$, and *d*, defined by *v/D* (where *v* is the speed of the vehicle and *D* is the correlation distance of the gravitational information), represents the reciprocal of the correlation time. Substituting Equation (18) into Equation (17) yields:

(19) $$W=\sqrt{2\sigma_{r}^{2}\left(1+\frac{e^{-t_{m}d}-1}{t_{m}d}e^{-t_{c}d}\right)}$$

Both Equations (17) and (19) show that the increase of $t_{m}$ or $t_{c}$ decreases the accuracy of compensation. When both of them become zero, *W* is also reduced to zero, which matches with the fact that in this case there is no discrepancy between the truth and actually used value. As $t_{m}$ and $t_{c}$ approach infinity, *W* is reaching its maximum $\sqrt{{2\Phi }_{r}(0)}$, which in the case of Equation (18) becomes $\sqrt{2}\sigma_{r}$.

**Figure 5:**
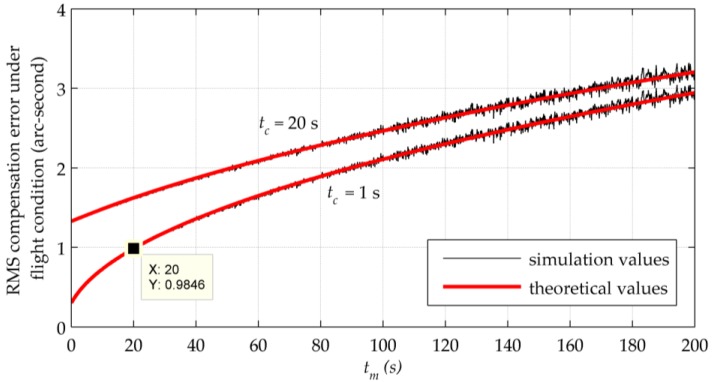
Simulation and theoretical values of the RMS difference between $x_{c}(t)$ and $x_{r}(t)$ under the common experimental flight condition.

According to the DOV data set released under the model EGM2008, the global arithmetic RMSs of the DOV are 5.417 (ξ) and 5.503 (η) arc-seconds [16,28]. Over the area of our sea test (whose scope will be described in Section 5), the arithmetic RMSs are 4.724 (ξ) and 7.404 (η) arc-seconds. In addition, the horizontal components of gravity disturbance vectors can be assumed to behave like the first-order Gauss-Markov stochastic process, whose autocorrelation is given as in Equation (18). The values chosen to fit the gravity field of the Texas-Oklahoma region is that $\sigma_{r}$ equals 21.8 mGal (around 4.59 arc-seconds) in both the along-track and cross-track directions, and that *D* equals 181 km for the along-track component and 838 km for the cross-track component. Thus, to produce a time requirement suitable for most occasions, a situation is assumed in which the DOV has a relatively big amplitude and changes quite drastically, and the values for $\sigma_{r}$ and *D* are assigned as 10 arc-seconds and 181 km, respectively. Considering the common experimental flight condition, the speed is assumed to be 80 m/s. Using these parameters, a set of simulated gravity disturbances was generated as $x_{r}(t)$ with $\sigma_{r}=10$ arc-seconds and $d\;=\;4.4199\times 10^{-4}$ to verify Equation (19). A series of $t_{m}$ covering the range from 0 s to 200 s was used to sample $x_{c}(t)$ from $x_{r}(t)$, with the time delay 1 s and 20 s respectively. Then the RMS difference between $x_{c}(t)$ and $x_{r}(t)$ was compared with the theoretical predication of Equation (19), as shown in Figure 5.

In Figure 5, simulation results show good agreements with theoretical values. The RMS difference grows with $t_{m}$, fast at the beginning and later approaching its steady state. On the other hand, the increase in $t_{c}$ shifts the entire RMS curve upward and makes it faster to approach the maximum $\sqrt{2}\sigma_{r}$. This effect is more notable when $t_{m}$ is smaller. The parameter $\sigma_{r}$ determines the amplitude and the upper limit of *W*. Moreover, the requirement of $t_{m}$ and $t_{c}$ for gravity compensation of the airborne INSs can also be concluded from Figure 5. If it takes more than 1 s to calculate the single-point DOV, there will be a very strict limit for the update interval. And if the calculation time is under 1 s, an update interval of 20 s can ensure the compensation error less than 1 arc-second, as what the marker shows in Figure 5.

**Figure 6:**
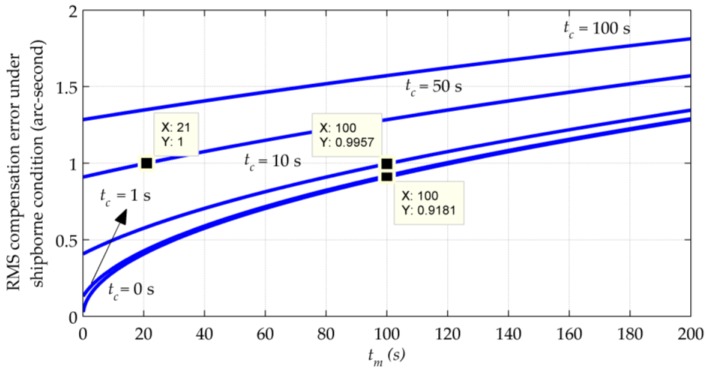
RMS compensation errors vs. update interval and computing time delay under the shipborne condition.

The requirement of $t_{m}$ and $t_{c}$ in marine navigation applications is also analyzed using Equation (19). The values of $\sigma_{r}$ and *D* remain unchanged, while the speed is chosen as 15 m/s, resulting in $d\;=\;{8.2873\times 10}^{-5}$. Figure 6 shows how RMS changes as a function of $t_{m}$ and $t_{c}$ under this circumstance.

Comparing Figure 6 with Figure 5, it can be seen that the growth of RMS compensation errors becomes slower as a result of a smaller *d*. As the error resulting from $t_{c}$ contributes a lot to the whole compensation error, it is still recommended that the calculation time should be no more than 1 s. Under this condition, using an update interval under 100 s can obtain a compensation accuracy better than 1 arc-second by a margin, as what the markers show in Figure 6.

In a word, higher speed, bigger amplitude and more drastic change of the DOV result in higher requirements on the update interval and computing time. In general, the actual gravity field changes more gently, and for the DOV the damped Schuler loop acts as a low-pass filter, both of which lead to longer correlation time and allow a longer update interval, but smaller computing time delay is still better. When the compensation error is required to be no more than 1 arc-second, it is recommended that the single-point DOV should be computed within 1 s and updated at an interval less than 100 s for marine navigation, and 20 s for airborne INSs.

### 4.2. Improvement of Computation Efficiency

Originally it took over 30 s to compute a gravity vector using the EGM2008 to degree 2190 and order 2159 with a desktop computer (Intel dual core processor i3-3240, 3.40 GHz, 3.40 GHz; physical RAM 3.41 GB available; 32-bit Windows 7 Professional; C language compiled by Microsoft Visual Studio 2010 Ultimate). Such a long time delay does not meet the time requirement and will result in unacceptable compensation errors.

Program profiling shows that most of the computation time are spent on locating and reading the spherical harmonic coefficients. The reason is that in the file provided by the EGM2008, the spherical harmonic coefficients stored in ASCII format records are first arranged by their corresponding degree *n* and then sub-arranged by their order *m*. Therefore, we removed the needless information and rearranged the coefficients in a binary file first by *m* and then by *n*. This modification matches with the modified forward column method to calculate the ALFs, and thus allows the program to read every coefficient sequentially just along with the recursion of ALFs and their first derivatives without the process of locating and transforming. In this way the average computing time of a gravity vector under the same computing environment has been shortened to less than 1 s, which makes it possible to calculate gravity vectors from the EGM2008 directly in situ. The size of necessary data is under 40 MB, which is much smaller than that of the original coefficient file (239.29 MB) or the high-resolution database for a large area (about 1 GB for global data gridded at 1 arc-minute). In addition, as the gravity vector can be calculated anywhere on and outside the Earth, both the interpolation and upward continuation, which bring errors when using ground databases, are no longer needed.

### 4.3. Compromise between Accuracy and Computing Efficiency

**Figure 7:**
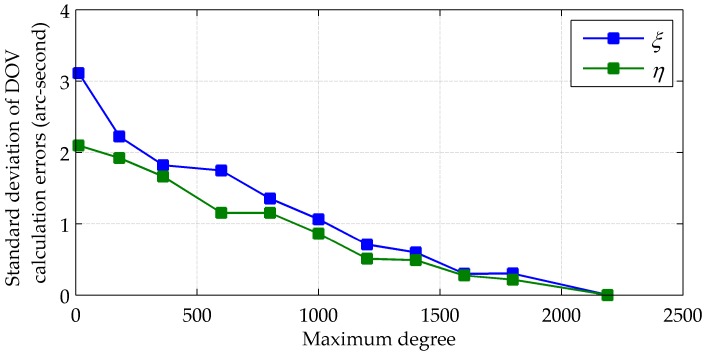
Calculation errors vs. maximum degree.

**Figure 8:**
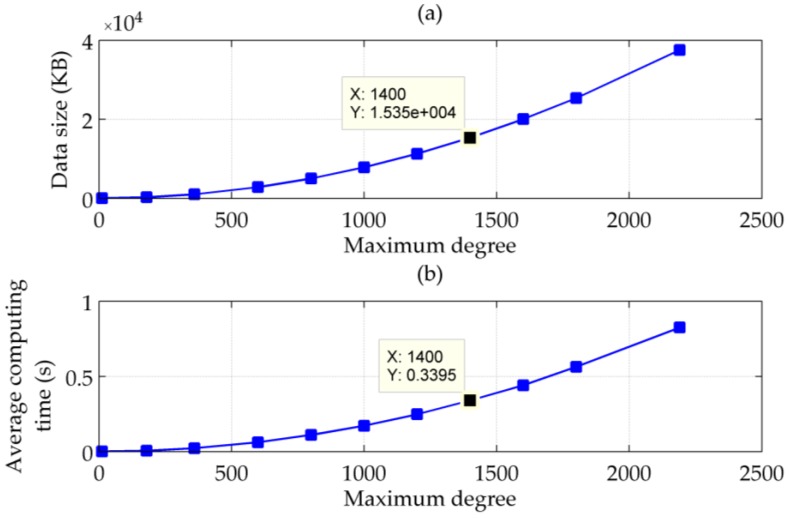
(**a**) Sizes of spherical harmonic coefficient files; and (**b**) Average computing time vs. maximum degree of the used spherical harmonic model.

If the maximum degree of the spherical harmonic model used to calculate the gravity vector is reduced, both the computing time and size of the coefficient file will decrease, but accompanied by a loss of detailed gravitational information and the non-gravitational artefacts. To find a compromise between the accuracy and computing efficiency, 4000 points on the route of the sea test are chosen to calculate their DOVs from EGM2008 to degree 12, 180, 360, 600, 800, 1000, 1200, 1400, 1600, 1800 and 2190 with matched coefficient files. Using the set of DOVs corresponding to degree 2190 as a reference, standard deviations of calculation errors of ξ and η are plotted in Figure 7, and changes of the average single-point computing time and the sizes of coefficient files are plotted in Figure 8. It can be seen that the maximum degree has to be bigger than 1000 to guarantee the calculation accuracy better than 1 arc-second. Taking into account the compensation errors resulting from the computing time and the update interval, the maximum degree should be no less than 1400, with a minimum average computing time within 0.4 s and a minimum file size less than 20 MB.

It should be noted that the results about truncation in this section are gained over the mid-latitude areas. Repeat tests were conducted in latitudes of 75°, 80° and 85°, over the longitude scope of 0°~180° with a discretization step of 0.1°.The results showed that the loss of accuracy becomes bigger near the pole and increases with the latitude. For example, when truncating the model at degree 1800, the differences become 2.07(ξ) and 1.84(η), 2.50(ξ) and 2.40(η), 2.78 (ξ) and 2.3(η) arc-seconds, respectively. This indicates that the gravity compensation around the polar areas needs more investigation, which could be one of our future research works.

Besides, although discussion in this part is aimed to provide some reference for systems with limited hardware resources, we suggest that the truncation of the model should be taken only as a last resort.

## 5. The Sea Test of a Shipborne INS

The shipborne INS used in the sea test is the same as in [6], where the specifications of the instruments are described in detail. Two dual-axis gyros with ultra-low drift and three orthogonal pendulous accelerometers are mounted on a gyro-stabilized gimbaled platform. An altimeter and a velocity log are used to provide the altitude and velocity reference for the damping of the vertical and horizontal channels. The high-order horizontal damping network used in the system has been introduced in Section 1. The position solutions of the INS are compared with the outputs of a GPS to obtain the real-time position errors.

**Figure 9:**
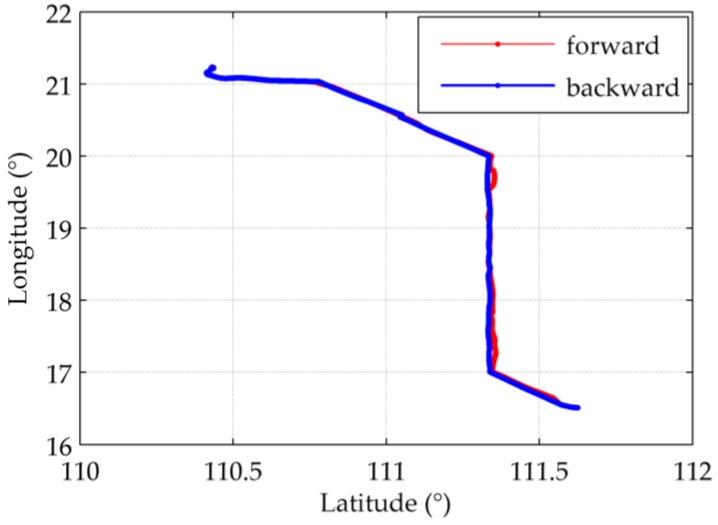
The route of a round-trip experiment during the sea test.

Some quite drastic changes in the original position errors were observed during the sea test, which does not match with the typical error propagation of the slowly varying INS errors. But they show a strong correlation with the ocean depths along the route, acquired from the 2 arc-minutes global relief model ETOPO2v2 released by the National Geophysical Data Center (NGDC). As a result, it is speculated that this anomalous phenomenon was caused by the gravitational errors, and a static experiment and a few dynamic experiments are conducted to compensate such gravity induced position errors. The route of a round-trip experiment is shown in Figure 9. Along this route several symmetrically distributed peaks can be observed in the original position errors, which implies some kind of relevance with the local underwater topography.

According to the coverage area of the sea test, we used the EGM2008 and Equations (10)–(12) to generate a 5 arc-minutes gridded local database of gravity vectors, covering latitude 5~25° N and longitude 105~120° E. The values of the gravity vectors were interpolated from this off-line database using the bilinear interpolation and were updated every 10 s. There will be a detailed discussion of the compensation results in the next section.

## 6. Results and Discussion

### 6.1. The Static and the Round-Trip Experiment

**Figure 10:**
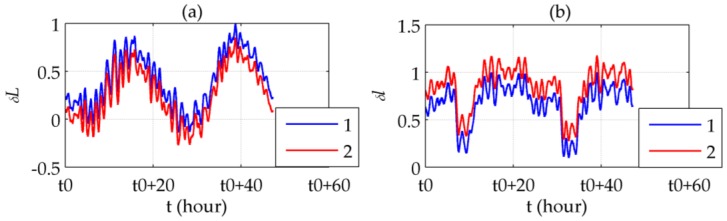
Results of the static experiment: (**a**) Latitude errors before (line 1) and after (line 2) compensation; (**b**) Longitude errors before (line 1) and after (line 2) compensation.

The static experiment was conducted during a period of anchoring, of which the compensation results is shown in Figure 10. All of the curves illustrated in Section 6 are normalized using the maximum absolute value of the uncompensated latitude or longitude errors in the corresponding segment. It can be observed that a constant offset exists between the position errors before and after compensation, which is almost the same as the corresponding components of the DOV at that position. The standard deviations of the difference between the compensated position errors and the corresponding components of the DOV are 2.38% of ξ in the latitudinal direction and 0.95% of η in the longitudinal direction.

**Figure 11:**
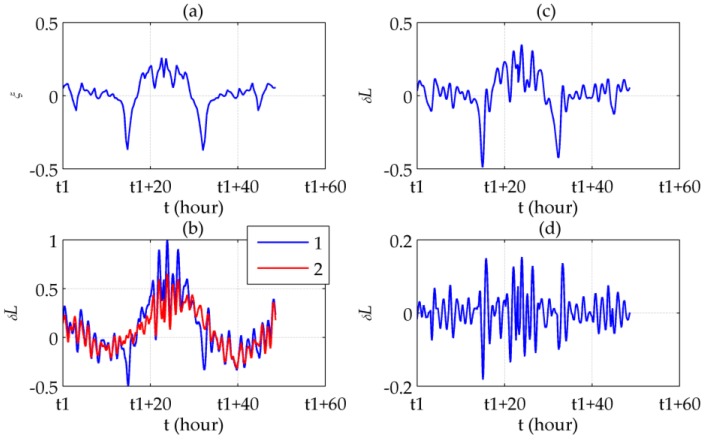
Latitudinal results of the round-trip experiment: (**a**) ξ; (**b**) Latitude errors before (line 1) and after (line 2) compensation; (**c**) Compensated latitude error; (**d**) Difference between ξ and the compensated latitude error.

The results of the round-trip experiment is shown in Figure 11 (the latitudinal direction) and 12 (the longitudinal direction), whose route has been shown in Figure 9. It locates at the very beginning of the sea test where the accumulation of INS errors has not become prominent.

**Figure 12:**
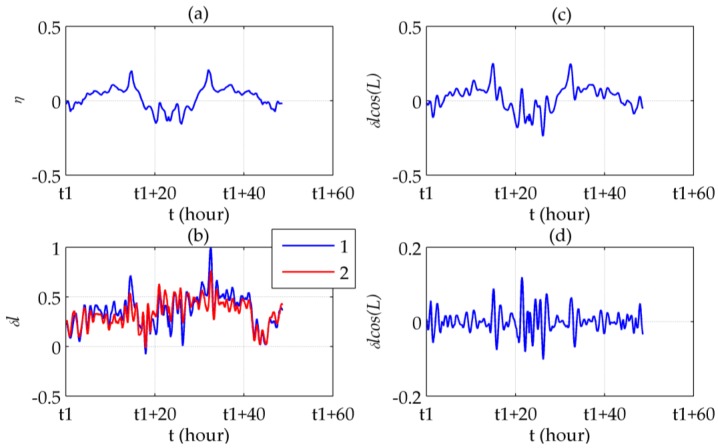
Longitudinal results of the round-trip experiment: (**a**) η; (**b**) Longitude errors before (line 1) and after (line 2) compensation; (**c**) Compensated longitude error; (**d**) Difference between η and the compensated longitude error.

Figure 11a,b shows that the symmetrically distributed peaks in δL appears where ξ reaches its peaks. Most of the peaks are removed after compensation, yielding a relatively steady 24-h periodic form which is typical for the long-term INS errors. Figure 11c illustrates the difference between the latitude errors before and after compensation, which is almost the same as ξ. The difference between the compensated errors and ξ represents the Schuler oscillation excited by ξ, as shown in Figure 11d. More intensely ξ fluctuates, the oscillation amplitude becomes bigger. Figure 12 illustrates similar results, except for using δlcos(L) instead of δl in Figure 12c,d according to Equation (8). The maximum error compensated is 48.85%, in which the Schuler oscillation takes 18.83%, of the maximum absolute value of the uncompensated horizontal position errors.

These results indicates that: (1) the vertical component of the gravity disturbance vector hardly affects the accuracy of INS solutions; (2) low-frequency components of the DOV will propagate into the latitude and longitude errors at a ratio of 1:1; and (3) fluctuations in the DOV excite a time-varying error response in the form of Schuler oscillation. All of them verifies the theoretical predication in Section 1 and the successful realization of the gravity compensation using EGM2008.

### 6.2. Dynamic Experiments after a Long-Time Navigation

As the inertial navigation has already lasted for quite a long time, the accumulation of INS position errors has become prominent enough to conceal the low-frequency components of the gravity induced errors. Over the areas where the topography changes drastically, we choose seven segments with time span around 10 h to observe the Schuler oscillation. In order to remove the 24-h periodical components, both the normalized latitude and longitude errors are fitted to quadratic polynomials. Time durations, scopes of corresponding latitude and longitude, and sum squared errors (SSEs) of the fittings, which evaluate the intensity of Schuler oscillation, are listed in Table 1.

**Figure 13:**
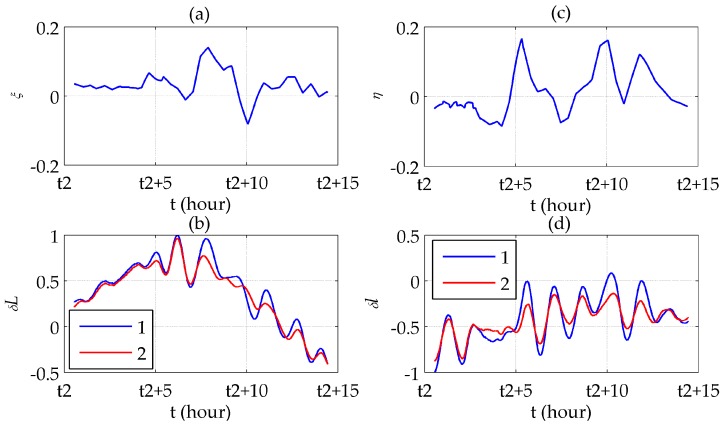
Compensation results of Segment II-5: (**a**) ξ; (**b**) Latitude errors before (line 1) and after (line 2) compensation; (**c**) η; (**d**) Longitude errors before (line 1) and after (line 2) compensation.

Here, the error curves of Segment II-5 are illustrated in Figure 13 as an example. All SSE values in Table 1 decreased after compensation, which indicates that the gravity induced Schuler oscillation has been attenuated. Besides, the more direct illustration in Figure 13 shows that the peaks of the DOV do not only cause larger errors at corresponding points, but also increase the nearby oscillation amplitude.

## 7. Conclusions

In the high-precision and long-term INSs with both the altitude damping and the horizontal velocity damping networks, the gravity disturbance vector induced errors exist almost exclusively in the horizontal channels and are mostly caused by the DOV. Low-frequency components of the DOV propagate into the latitude and longitude errors at a ratio of 1:1. Moreover, the fluctuations in the DOV excite Schuler oscillation since the system is underdamped. To compensate these errors, two methods based on the EGM2008 are provided in this paper, namely, interpolation from an off-line database generated beforehand using the spherical harmonic model and computing the values of gravity vectors from the model directly in situ.

A formula is developed to characterize the relationship of the update time interval, the computing time delay and their resulting compensation errors, which produces a time requirement for the real-time gravity compensation in INSs. Typically, it is recommended that the gravity vector should be calculated within 1 s and update at an interval less than 100 s for the marine navigation, and 20 s for the airborne INSs, to ensure the compensation accuracy better than 1 arc-second. After optimizing the layout of spherical harmonic coefficients, the average single-point computing time has been reduced greatly to less than 1 s, which makes it possible to implement the second method for real-time gravity compensation applications.

Several off-line compensation experiments were conducted using the data of a high-precision shipborne INS and auxiliary test instruments collected during an actual sea test. With the aid of EGM2008, both low-frequency components and Schuler oscillation of the gravity induced position errors are attenuated, up to 48.84% in total of the regional maximum in the rugged terrain. The experimental results agree well with the theoretical predication, and indicate that the EGM2008 has enough accuracy and resolution for the gravity compensation in such high-precision long-term INSs.

It should be noted that the sea test is conducted in regions with unrestricted gravity anomaly data during the development of EGM2008. As for areas where gravity anomaly data are unavailable, such as Antarctica, more tests will be needed to further investigate the EGM2008’s performance in gravity compensation. Our future work will focus on the further improvement of gravity compensation accuracy over such regions. For example, the model GGMplus with ultra-high resolution will be taken into consideration in the possible future flight test over land areas.

## Figures and Tables

**Table 1:** SSEs of fittings of the normalized horizontal position errors before and after compensation.

No.	II-1	II-2	II-3	II-4	II-5	II-6	II-7
Time span (h)	7.22	10.28	10.55	6.66	13.88	7.22	7.50
Latitude scope (°)	13.15~14.65	10.07~10.44	9.80~10.21	9.60~9.85	13.41~15.51	13.96~15.10	14.05~15.16
Longitude scope (°)	115.52~115.61	114.21~115.34	114.23~115.54	114.64~115.53	112.33~112.52	112.35~112.44	112.30~112.43
Uncompensated δL SSE	62.01	65.95	92.88	33.06	78.33	36.52	39.25
Compensated δL SSE	12.41	26.57	49.09	13.49	34.27	9.66	19.19
Uncompensated δl SSE	34.89	75.69	367.66	384.84	187.18	236.06	58.58
Compensated δl SSE	20.65	33.21	157.40	99.80	66.22	81.99	15.88
